# Engineering Molecular Beacons for Intracellular Imaging

**DOI:** 10.1155/2012/501579

**Published:** 2012-11-06

**Authors:** Cuichen Sam Wu, Lu Peng, Mingxu You, Da Han, Tao Chen, Kathryn R. Williams, Chaoyong James Yang, Weihong Tan

**Affiliations:** ^1^Center for Research at Bio/Nano Interface and Department of Chemistry and Department of Physiology and Functional Genomics, Shands Cancer Center, UF Genetics Institute and McKnight Brain Institute, University of Florida, Gainesville, FL 32611-7200, USA; ^2^State Key Laboratory of Physical Chemistry of Solid Surfaces and Department of Chemical Biology, Key Laboratory of Analytical Science, College of Chemistry and Chemical Engineering, Xiamen University, Xiamen 361005, China

## Abstract

Molecular beacons (MBs) represent a class of nucleic acid probes with unique DNA hairpin structures that specifically target complementary DNA or RNA. The inherent “OFF” to “ON” signal transduction mechanism of MBs makes them promising molecular probes for real-time imaging of DNA/RNA in living cells. However, conventional MBs have been challenged with such issues as false-positive signals and poor biostability in complex cellular matrices. This paper describes the novel engineering steps used to improve the fluorescence signal and reduce to background fluorescence, as well as the incorporation of unnatural nucleotide bases to increase the resistance of MBs to nuclease degradation for application in such fields as chemical analysis, biotechnology, and clinical medicine. The applications of these *de novo* MBs for single-cell imaging will be also discussed.

## 1. Introduction

Over the past decade, the molecular processes inside cells have been intensively investigated, including, for example, translocation of proteins and the dynamics of transcription and translation, directly affecting the fields of molecular cell biology, drug discovery, and medical diagnostics [1]. The key to the effective and successful monitoring of single-cell dynamics is the development of ultrasensitive and quantitative imaging with specific recognition of targets in living cells. To accomplish this, various nucleic acid (NA) probes, in particular, molecular beacons, have been proposed on the basis of their facile synthesis, unique functionality, molecular specificity, and structural tolerance to various modifications [2]. Since the first report of MBs in 1996 [3], they have become widely used for real-time observation of RNA distribution and dynamics in living cells. 

**Figure 1:**
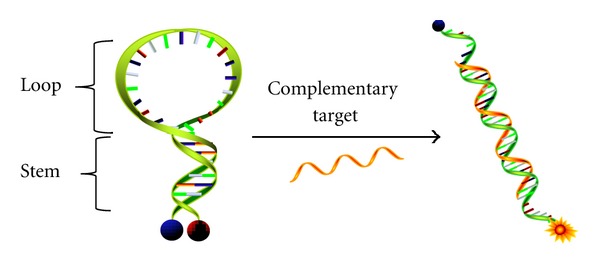
Schematic design of a molecular beacon. Hairpin-shaped MBs have a fluorophore (orange) and a quencher (blue) on the 5′ and 3′ ends, respectively. In the absence of target sequences, the fluorescence of MBs is quenched due to the close proximity between the fluorophore and quencher. After introduction of the complementary sequence, the cDNA will force the stem helix to open, resulting in a fluorescence restoration [4].

As shown in Figure 1, molecular beacons are hairpin-shaped oligonucleotides with a fluorescence donor on one end and an acceptor on the other end. Generally, molecular beacons are composed of a 15–30 base loop region for target recognition and a double-stranded stem containing 4–6 base pairs. The signal transduction mechanism of molecular beacons is mainly based on fluorescence resonance energy transfer (FRET). A fluorescence donor in the excited state transfers the absorbed energy to a nearby fluorescence acceptor *via* dipole-dipole coupling, causing fluorescence emission by the acceptor and/or quenching of fluorescence donor. Because the efficiency of energy transfer is significantly affected by the distance between the donor and the acceptor, the decrease in donor fluorescence and/or the increase in acceptor fluorescence can be used to study the binding events between a single-strand nucleic acid and its target. Therefore, in the absence of target DNA, RNA, or protein, molecular beacons maintain the loop-stem structure, resulting in quenching due to the close proximity between fluorescence acceptor and donor (OFF state). However, upon target binding, a spontaneous conformational change occurs to open the stem and restore the fluorescence signal (ON state). By monitoring the change of fluorescence intensity, molecular beacons have been used for the detection of DNA and RNA in living systems [3, 5–7], design of biosensors [8, 9], and investigation of protein-DNA interactions [10–12]. 

After nearly two decades of development, MBs have attracted interest for real-time intracellular monitoring based on their unique properties, including, for instance, possibility of RNA detection without the need to separate the bound and unbound probes, high sensitivity, and the selectivity required to differentiate between sequences with single-base mismatches [4]. However, when applied in intracellular environments, MBs continue to be hindered by: (1) low signal intensity from a single fluorophore and vulnerability to photobleaching, which limit sensitivity; (2) unquenched high background signal from the MB itself, which causes limited increase of the signal-to-background ratio upon target binding; (3) tendency toward instability in living cells by the degradation by endogenous nucleases and nonspecific binding of cytoplasmic proteins, events which result in false-positive signals. To solve these problems, molecular engineering of MBs has been introduced using, for instance, water-soluble conjugated polymers (CPs) [13] and artificial nucleotides, such as locked nucleic acid (LNA) [14] and l-DNA [15], as well as molecular assembly of an array of quencher molecules to produce superquenchers (SQs) [16] or hybrid molecular probes (HMPs) [17]. This paper will first describe the recent developments in molecular engineering that improve MBs for use in intracellular imaging, including increasing signal intensity, reducing interfering background fluorescence, and enhancing biostability. This will be followed by a discussion of how these newly engineered MBs are applied in intracellular imaging to achieve simultaneous monitoring of target molecules. 

## 2. Molecular Engineering of Molecular Beacons

### 2.1. Conjugated Polymer (CP-) Modified MBs to Increase Fluorescence Signal

**Figure 2:**
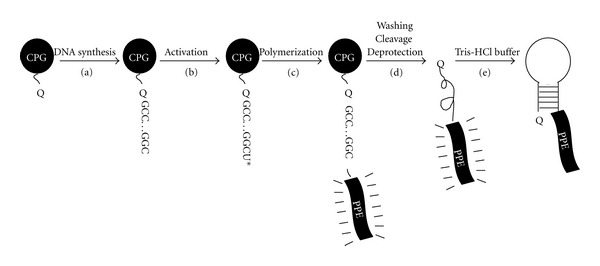
Schematic solid state synthesis procedure of PPE-labeled molecular beacons [13]. (Q: DABCYL quencher).

Conjugated polymers (CPs) are polyunsaturated macromolecules in which all backbone atoms are sp- or sp^2^-hybridized. They are known to exhibit photoluminescence with high quantum efficiency [18]. A unique and attractive property of fluorescent CPs is their fluorescence superquenching effect [19, 20], allowing a hundred- to a millionfold more sensitivity to fluorescence quenching compared to that of their low molecular weight analogues. Among these CPs, water-soluble poly(phenylene ethynylene)s (PPEs) are particularly attractive candidates for optical biosensing applications by their high fluorescence quantum yields in aqueous solutions [21]. PPEs can be prepared through palladium (Pd) catalyzed cross-coupling of bisacetylenic and diiodoaryl monomers in an amine environment [22]. After synthesis of MBs on a DNA synthesizer through solid phase phosphoramidite chemistry, a 5I-dU residue is introduced into each MB as a monomer of polymerization, followed by cross-coupling of the polymer chain with MBs (Figure 2) [13]. Based on the superquenching property of the conjugated polymer, the CP-modified MBs have greatly amplified the signal/background ratio compared to traditional MBs. 

### 2.2. Reduction of Background Fluorescence

Although MBs are designed for their specific complementary targets, incomplete quenching can occur due to a variety of reasons. First, the probe itself cannot be perfectly quenched even by the close proximity of acceptor and donor, thus limiting signal enhancement. Second, false-positive signals arise from degradation by nucleases or nonspecific binding of proteins. Third, traditional molecular beacons easily suffer from interruption of the stem structure. This can be explained by: (1) the complicated cellular environment, in which chances abound for undesired intermolecular interactions between stems and their complementary sequences or (2) the thermodynamic conformational switch between hairpin and nonhairpin structures. To address the problem of high background fluorescence, the Tan group has adopted a variety of successful strategies, as discussed in this section. 

**Figure 3:**
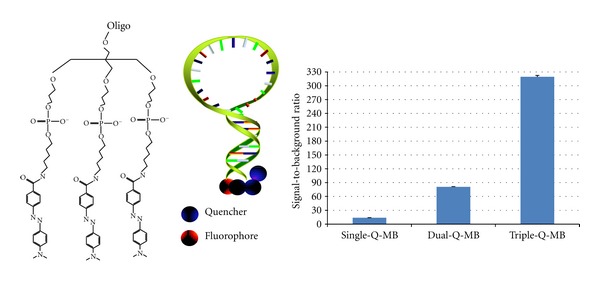
Schematic of a molecular beacon conjugated with a superquencher consisting of triple DABCYLs. The signal-to-background ratio of molecular beacons increases as the number of quenchers increases [16].

To improve the signal-to-background ratio of MBs, the most straightforward method involves increasing the number of quenchers. By the molecular assembly of different numbers of quenchers on one end of MBs, while keeping only one fluorophore on the other end, Yang et al. achieved high sensitivity and specificity [16]. Multiple quenchers improve absorption efficiency and increase the probability of dipole-dipole coupling between the quenchers and fluorophore (Figure 3). The quenching efficiency of DABCYL increased as the number of DABCYL moieties increased: 92.9% for single DABCYL, 98.75% for dual DABCYLs, and 99.7% for triple DABCYLs, as a superquencher (SQ). Such superquencher MB assemblies demonstrated a 320-fold fluorescence enhancement upon a target binding, a significant improvement compared to a single-quencher with only 14-fold enhancement. Superquencher-labeled MBs showed great sensitivity, higher thermal stability, and slightly improved specificity compared to regular MBs. This strategy can also be used for other nucleic acid probes, such as aptamers [23–25], which generated a 49000-fold signal increment when PDGF aptamers bound to PDGF proteins. 

**Figure 4:**
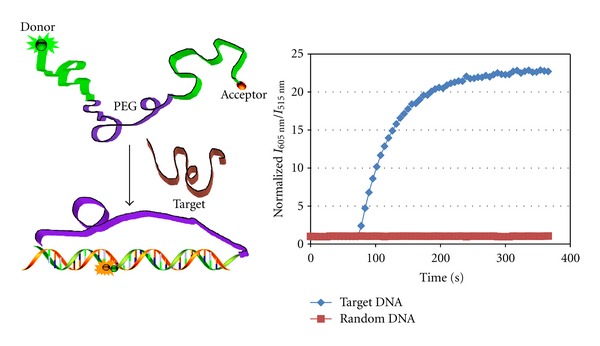
Working principle of hybrid molecular probe (HMP) binding to target nucleic acid sequence. Fluorescence kinetic study of HMP to the target and control sequence [17].

Negative signals of molecular beacons typically result from sticky-end pairing between hybridized MBs [26]. This was solved with the introduction of the hybrid molecular probe (HMP) developed by Yang et al. [17]. Two single-stranded DNA sequences, each complementary to part of the target DNA, were linked by a flexible poly(ethylene glycol) (PEG) spacer. The fluorescence acceptor and donor moieties are labeled on each terminus (Figure 4). Upon hybridization to target, the 5′ and 3′ ends of the HMP are brought into close proximity, resulting in a fluorescence resonance energy transfer (FRET) signal. False-positive signals due to nucleases and nonspecific binding to proteins were greatly reduced even in cancer cell lysate. Compared to conventional MBs, HMPs have intrinsic advantages. First, its special loop-stem structure, which is based on the sequence of target NA, is easier to design. Second, while MBs are hindered by the energy barrier of the self-complementary stem structure, which slows down hybridization kinetics, HMPs respond to target DNA/RNA more rapidly due to the absence of stem structures in HMP. Third, although unmodified MBs cannot avoid false-positive signals or nonspecific protein binding, HMP can easily overcome these obstacles by linking two oligonucleotides with a PEG spacer.

**Figure 5:**
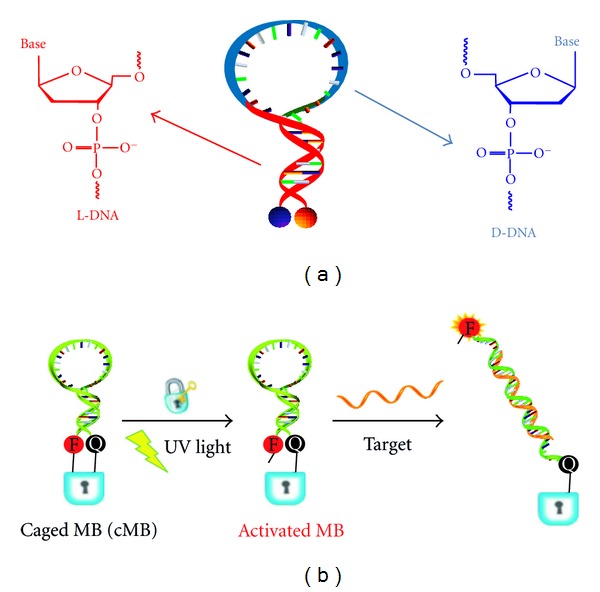
(a) Schematic of molecular beacon using l-DNA for the stem part (red) and d-DNA for the loop part (blue) [15]. (b) Principle of caged molecular beacons (cMBs) locked by covalent bonding or biotin-avidin interaction *via* photocleavable linkage. After light illumination, activated MBs will recover the hybridization to complementary target [27].

Incorporation of unnatural enantiomeric l-DNA in the stem of a molecular beacon is another strategy to prevent the occurrence of false-positive signals caused by the undesired intermolecular interactions between stems and their complementary sequences (Figure 5(a)) [15]. While l-DNA and d-DNA have identical physical properties, they cannot form stable duplex structures as expected for d-DNA complementary strands. MBs with d-DNA loop and l-DNA stem have better sensitivity and stability, for example, higher signal-to-background ratio and melting temperature. More importantly, MBs with l-DNA-modified stems can effectively prevent false-positive signals caused by nonspecific hybridization of d-DNA sequences to the stems of conventional MBs with d-DNAs. 

Incomplete quenching can also be checked by locking the stem of a molecular beacon with a photo-labile molecular interaction or covalent bond. Without light irradiation, the light-activatable MBs are inactive, even in the presence of target sequence. After unlocking with a quick light illumination, the decaged MBs recover their ability to hybridize to complementary DNA/RNA. Inspired by this design, Wang et al. made use of a biotin-avidin interaction or triazole to lock the stem of MBs *via* a photocleavable linker (PC linker) bearing an *o*-nitrobenzyl moiety [27]. The cMBs have lower background fluorescence based on the tighter distance between fluorophore and quencher that results from the covalent linkage or high affinity interaction in the stem part (Figure 5(b)). This photocaged technique will find wide application in the study of gene expression, protein synthesis, and cell signaling with high temporal and spatial resolution. 

**Figure 6:**
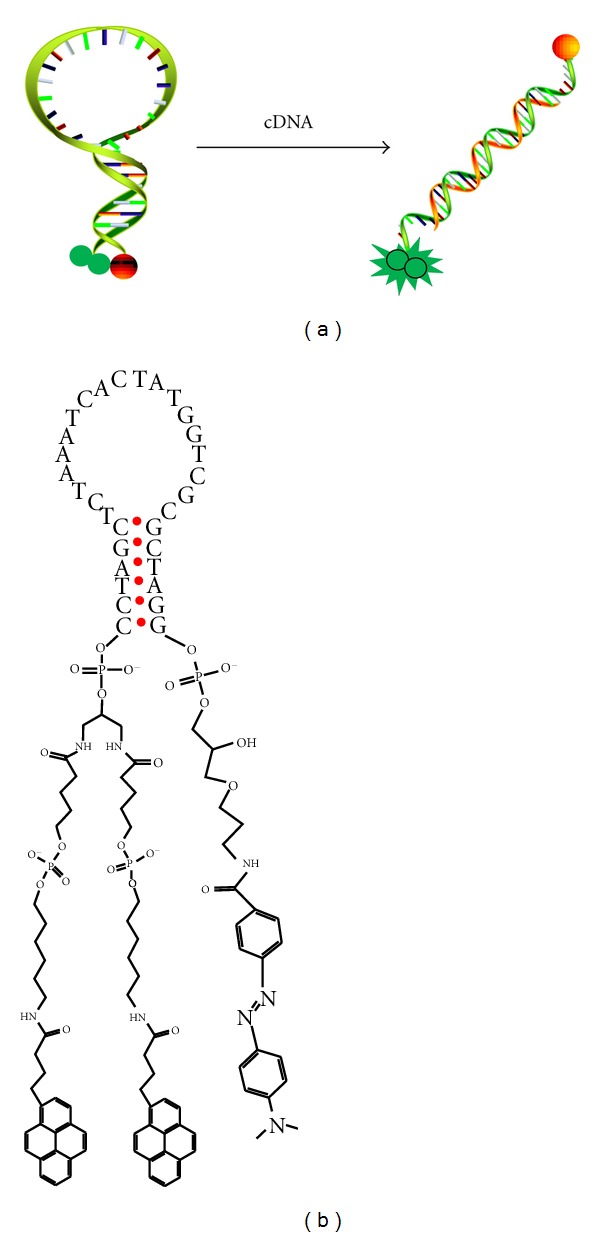
(a) Scheme of a dual pyrene-labeled molecular beacon hybridized with complementary target sequence (green ball = pyrene; red ball = DABCYL quencher). (b) Chemical structure of dual-pyrene-modified molecular beacon with pyrene monomer and DABCYL on the 5′ and 3′ ends, respectively [28].

Apart from the molecular probe itself, significant background interference also arises from the native fluorescence in complex biological fluids. Species in the physiological environment can have a strong autofluorescence background, which may reduce the sensitivity of NA probes. To address this issue, Yang et al. molecularly engineered NA probes with a spatially sensitive fluorescent dye, such as pyrene, to monitor proteins, RNA, and small molecules in complex biological environments [28–30]. Excited state dimers (excimers) are formed when an excited-state pyrene encounters a ground state pyrene [31]. The excimer emission is a broad, featureless band centered at 480 nm to 500 nm, which can be easily differentiated from the pyrene monomer that emits in the range from 370 nm to 400 nm. The excimer also has a very long fluorescence lifetime compared to other potential fluorescent species (as much as 100 ns or longer), while most biological background species have lifetimes of at most 5 ns. In the case of pyrene-labeled MBs, varied numbers of pyrene molecules are conjugated on the 5′ end of the MB sequence (Figure 6) [28]. In the absence of complementary DNA, the fluorescence of the pyrene monomer and excimer is quenched by the close proximity of pyrenees and DABCYL. However, the pyrene excimer fluorescence is restored after introduction of cDNA, which induces opening of the loop and hence, separates the pyrenees from DABCYL. Compared to FAM-labeled MBs, MBs labeled with multiple pyrenees have higher signal enhancement after addition of equimolar target. More importantly, time-resolved fluorescence was able to differentiate the fluorescence signal from the pyrene-labeled probe and complex biological species, for example, cell growth media. During the first 10 ns, the excimer emission spectra were hidden by the severe background fluorescence from cell media, similar to the emission spectrum of steady-state measurement. However, because of the different lifetimes among pyrene excimer, pyrene monomer and background fluorescence, the signal from pyrene excimer emission could be differentiated from the intense background interference 40 ns after the excitation pulse. In the chosen time window, much of the excimer emission still occurred, while most of the background autofluorescence had decayed [28, 30]. Using time-resolved methods, multiple pyrene-labeled MBs have the potential for sensitive measurement of low nanomolar target DNA in complex biological environments. 

### 2.3. Biostability Enhancement

**Figure 7:**
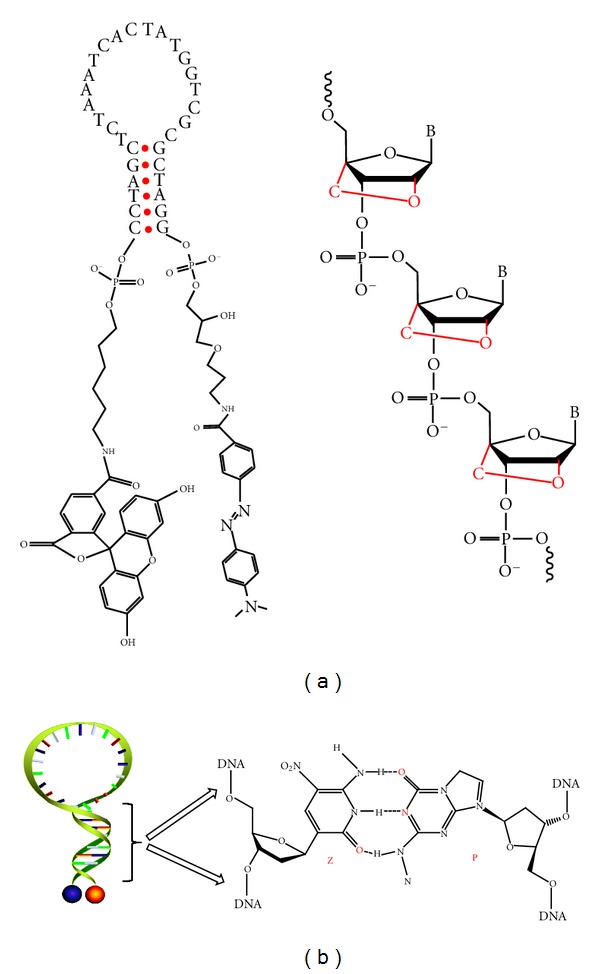
(a) Chemical structure of a molecular beacon and a LNA sequence [32]. (b) Schematic of MBs with a dZ : dP-modified stem [33].

Intracellular nuclease degradation and nonspecific protein binding thwart the use of traditional NA probes. To solve this problem, many chemically modified nucleotides have been proposed to increase the biostability of molecular beacons and prevent false-positive signals. For example, Wang et al. designed a molecular beacon using a locked nucleic acid (LNA) base, which has a methylene bridge connecting the 2′-oxygen of the ribose and the 4′-carbon (Figure 7(a)) [14]. The LNA possesses unique properties relative to a normal nucleotide. First, the LNA-LNA duplex has tighter binding and maintains s stable structure at 95°C. Second, LNA MBs are superior to DNA MBs in discriminating single-base mismatches. Finally, LNA MBs can resist interference by nonspecific proteins, such as single-stranded DNA binding protein (SSB) and the degradation by nucleases in the cell environment. However, the hybridization kinetics of LNA MBs are relatively slow compared to DNA MBs. Therefore, Yang et al. synthesized DNA/LNA chimeric MBs, which significantly improved the hybridization rates and maintained resistance to nonspecific protein binding and nuclease digestion [32]. 

Artificial nucleotides, which rely on an artificially expanded genetic information system (Aegis), have also been used to design molecular beacons. Sheng et al. synthesized 6-amino-5-nitro-3-(1′-*beta*-d-2′-deoxyribofuranosyl)-2(1H)-pyridone (d**Z**) and 2-amino-8-(1′-*beta*-d-2′-deoxyribofuranosyl)-imidazo [1,2-*a*]-1,3,5-triazin-4(8H)-one (d**P**) as the Aegis pair and incorporated this pair into the stem part of MBs (Figure 7(b)). The d**Z** : d**P **pair-modified MBs have excellent enzymatic resistance compared to normal MBs, as well as a hybridization interaction stronger than that of the dC : dG pairs, which provides the potential for effective discrimination against mismatched bases in short DNA duplexes [33]. 

### 2.4. Molecular Beacon Functionalized Nanomaterials

The rapid development of nanotechnology further facilitates the wide application of molecular beacons in disease diagnosis [34], biological visualization in living cells [35], and measurement of ribozymal catalytic activity [36]. The unique chemical and physical properties of nanomaterials, especially gold nanoparticles (AuNPs), including easy preparation, precise control of size and shape, facile modification with different ligands, and the binding-induced alteration in surface plasmon resonance, conductivity, or redox behavior [37], as well as the excellent biocompatibility and delivery efficiency into living cells [38–40], make metal nanomaterials outstanding candidates for the design of biosensors and molecular imaging. To solve the issue of molecular beacons in high instrument cost and requirement of well-trained operators, Mao et al. developed a dry-reagent strip-type nucleic acid biosensor (DSNAB) based on the assembly of MB-modified gold nanoparticles and a lateral flow test strip [34]. A DSNAB device consists of a sample pad, a conjugate pad for the specific hybridization between target DNAs and biotin-labeled MB-AuNPs, a nitrocellulose membrane with one test line and other control line, and an absorption pad. When an unknown sample solution with target DNA is applied on the sample pad, it starts to migrate to the conjugate pad by capillary action. Target DNA opens the MB hairpin structure resulting in activation of biotin on the area of conjugate pad, followed by the binding between these activated biotin-labeled MBs and preimmobilized streptavidin in the test line, causing an intense red band. The excess biotin MB-AuNPs are captured in the control zone causing another red band. Without target DNAs in unknown solution, only one red band can be observed at the control line, which shows that the device is functioning properly. This low-cost and sensitive detection device was able to achieve a detection limit of 50 pM nucleic acids with a portable strip reader in 15 min. 

Another strategy to overcome the photobleaching and photodegradation of organic fluorophores in molecular beacons is preparation of MB-quantum dot conjugates, because semiconductor quantum dots (QDs) are brighter and more resistant to photobleaching than organic fluorophores [41]. Yeh et al. developed AuNP-modified nuclease-resistant MBs and quantum dot hybrid nanoprobes for real-time visualization of virus replication in living cells [35]. The fluorescence emission of quantum dot is quenched by almost 100% by nearby gold nanoparticles. But hybridization with the viral genome in coxsackievirus (CVB6-) infected Buffalo green monkey kidney (BGMK) cells moves the AuNPs away from the quantum dots, and the QD fluorescence signal is restored. This nanoprobe can be taken into viral infected cells to monitor newly synthesized viral RNA in real time. 

In another application, a nanometal surface energy transfer (NEST) method was employed as a molecular ruler to analyze the conformational changes of hammerhead ribozymes in real time. The NEST method was preferred to conventional FRET due to the larger energy transfer distance in NEST [36]. The hammerhead ribozyme has a core loop flanked by three stems (stems I, II, and III) after binding with substrate. Jennings et al. modified a 1.4 nm AuNP at the 5′ end of the ribozyme and a FAM fluorophore at the 5′ end of the substrate to monitor the distance changes during different steps of the ribozyme-catalyzed reaction (binding between ribozyme and substrate, folding and final cleavage). The results showed separation distances of 6.6 ± 0.9 nm and 4.5 ± 0.8 nm between the FAM fluorophore and gold nanoparticle for relaxed and activated hammerhead complexes, respectively, confirmed by the classical FRET measurement of 6.3 ± 0.4 nm and 4.9 ± 0.3 nm. Furthermore, the rate constants of ribozyme binding to substrate and cleavage were 14.9 ± 1.5 *μ*M^−1^ min^−1^ and 0.013 ± 0.001 min^−1^, respectively, in close agreement with previous reports. 

## 3. Molecularly Engineered Probes for ****Intracellular Imaging

Since the first report of molecular beacons for intracellular real-time monitoring of RNA by Tyagi and Kramer in 1996, numerous types of MBs have been developed for target measurement in living cells without the need for separation of unbound probes or additional signal amplification. As a result of cell-to-cell variation, intracellular imaging with MBs has usually employed the strategy of ratiometric measurement, whereby one MB was designed for a specific target of interest, and the other served as the reference probe. Drake et al. investigated the stochasticity of human manganese superoxide dismutase (MnSOD) mRNA expression in breast cancer cells using a molecular beacon that targeted MnSOD mRNA, while the reference MB targeted *β*-actin mRNA [42]. A Ru(Bipy)_3_^2+^-labeled scrambled DNA sequence was used as a negative control. Lipopolysaccharide (LPS) is an inflammatory mediator involved in *Escherichia coli* bacterial sepsis and is proven to stimulate MnSOD mRNA expression in multiple mammalian cells. After LPS treatment, the MnSOD mRNA expression level in the MDA-MB-231 cell line, as detected by MnSOD mRNA MB, showed a distinct cell-to-cell variation, that is, 0.57 ± 0.17 to 0.66 ± 0.18 on average. On the other hand, for the *β*-actin MB, LPS treatment showed very little change relative to cell distribution, either before or after LPS induction. 

**Figure 8:**
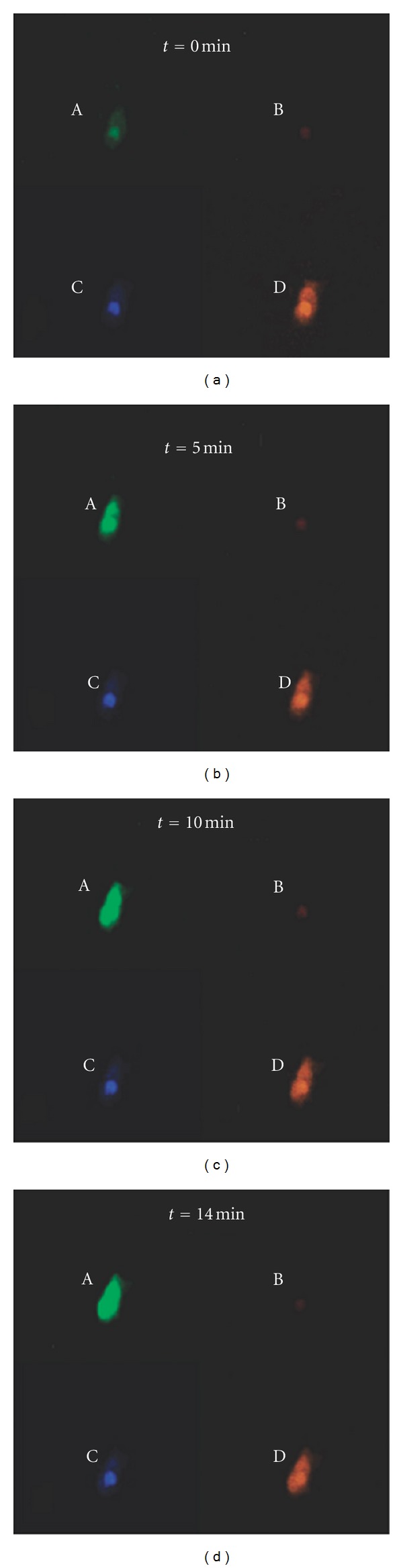
Real-time monitoring of multiple gene expression using different MBs in a single MDA-MB-231 cell. (a) Fluorescence imaging of the *β*-actin MB (green), (b) fluorescence imaging of control MB (red), (c) fluorescence imaging of MnSOD MB (blue), and (d) fluorescence imaging of Ru(Bpy)_3_^2+^ reference probe (orange) [43].

In addition to probing one pattern of cancer-related mRNA expression, MBs can also monitor multiple gene expression in a single living cell. Medley et al. synthesized three MBs labeled with different fluorophores to monitor the expression level of human MnSOD and *β*-actin mRNA in a single MDA-MB-231 cell [43]. Ru(Bipy)_3_^2+^ was chosen as the label for the reference probe in channel D, due to its stable emission fluorescence intensity and lack of fluorophore crosstalk. After microinjection, in channel B only a small amount of fluorescence signal was observed for control MB, which was designed to have no target complementary mRNA inside the cell. At *t* = 0 min, a fluorescence signal was only barely observed for *β*-actin MBs; however, the fluorescence intensity increased as time elapsed, consistent with the high expression of *β*-actin in the MDA-MB-231 cell. In channel C of MnSOD MBs, the fluorescence intensity showed the same increasing trend but not to the extent seen in the *β*-actin MBs (Figure 8). Furthermore, the varied pattern of gene expression in a single cell can be determined by this method. LPS induction showed a significant impact on the expression of MnSOD relative to that of *β*-actin. Besides probing multiple gene expression, Medley et al. also applied MBs and a cell-permeant Fluo-4 calcium ion indicator to investigate both mRNA expression levels and ion concentrations and their relationships in the same living cell [44].

**Figure 9:**
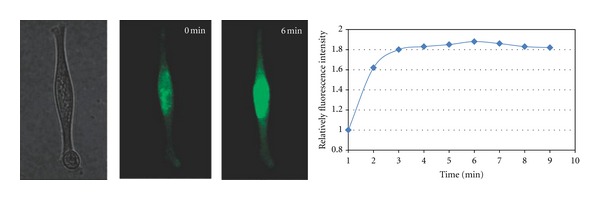
Time-lapse confocal fluorescence images of control MBs injected in a single cell for 24 h. At *t* = 0 min, excess amount of cDNAs of control MBs was microinjected into the cell [46].

To avoid interference from false-positive signals, for example, nonspecific protein binding and nuclease degradation, Martinez et al. used the HMP for intracellular studies of mRNA expression levels, as discussed in Section 2.2 [45]. Compared to MBs, HMPs have faster hybridization kinetics and greater resistance to nuclease degradation inside cells. Multiple mRNA sequences, such as *β*-tubulin, *β*-actin, and MnSOD, were chosen as targets for the design of three HMPs labeled with different fluorophores. After introduction into single cells by microinjection, the HMPs showed an intense FRET signal when hybridized to target mRNA, while the control HMP without cellular target showed only the signal of the fluorescence acceptor. This work indicated that HMPs had far less propensity for false-positive signals and performed better than traditional MBs inside living cells. The lifetime of molecular beacons in living cells is usually ~30 min, and after that, MBs will be digested by cellular nucleases and show false-positive signals. Therefore, investigators must address this problem if prolonged long-term real-time monitoring in single living cells is to be achieved. Inspired by locked nucleic acids (LNAs) [14, 32], Wu et al. developed LNA/DNA MBs with LNA-modified loops and LNA-/DNA-mixed stems to monitor mRNA expression in real-time for 5–24 h [46]. During treatment with LPS for 4 h, target MnSOD MBs showed a distinct increase in fluorescence intensity, while no change in the confocal fluorescence imaging was observed for control MBs. After injection into living cells over 24 h, the control MBs still retained their function and showed an intense fluorescence signal after introduction of their complementary target (Figure 9).

## 4. Conclusion

Nucleic acid probes, especially molecular beacons, have been increasingly developed for intracellular imaging of RNA, proteins, and small molecules over the last two decades. Based on their unique properties, including high sensitivity and selectivity for quantitative investigation of gene expression, as well as detection without separation of unbound probes inside cells, MBs have become an ideal molecular tool widely used in chemistry, biology, biotechnology, and medical science for biomolecular recognition [2, 4, 47–52]. More research is expected in three areas. First, traditional strategies of introducing MBs, such as microinjection, are time consuming and rely on the operator's skill. Other methods, like cationic polymers, suffer from toxicity and poor delivery efficiency. Therefore, development of safe and efficient delivery methods for MBs is essential for accurate measurement of gene expression in a large population of cells. Second, detection sensitivity of intracellular imaging in living cells should also be considered. Recently, a series of novel nucleic acid probes conjugated with nanomaterials, for example, graphene oxide (GO), have been developed to improve the detection limit [53, 54]. Third, and most challenging, is intracellular quantitative imaging of RNA, protein, and small molecule dynamics, which requires the implementation of new molecular probes and methodologies. Although many challenges remain, the work so far in the modification of molecular beacons and their application has resulted in advances in intracellular imaging that could not have been anticipated only ten years ago.
